# A multiplex PCR assay for the identification of fruit flies (Diptera: Tephritidae) of economic importance in South Africa

**DOI:** 10.1038/s41598-022-17382-x

**Published:** 2022-07-29

**Authors:** Kelsey J. Andrews, Rachelle Bester, Aruna Manrakhan, Hans J. Maree

**Affiliations:** 1 Department of Genetics, Stellenbosch University, Private Bag X1, Matieland, 7602 South Africa; 2 Citrus Research International, PO Box 2201, Matieland, 7602 South Africa; 3 Citrus Research International, PO Box 28, Mbombela, 1200 South Africa; 4 Department of Conservation Ecology and Entomology, Stellenbosch University, Private Bag X1, Matieland, 7602 South Africa

**Keywords:** Entomology, PCR-based techniques

## Abstract

The fruit fly (Diptera: Tephritidae) species, *Ceratitis capitata*, *Ceratitis cosyra*, *Ceratitis rosa*, *Ceratitis quilicii*, and *Bactrocera dorsalis* are of economic importance in South Africa. These agricultural pests cause extensive damage to a range of commercially produced fruit, primarily for export. These pests are of phytosanitary significance, and their presence in fruit-producing regions in South Africa has led to restrictions in export trade of fresh produce. Accurate identification of these flies, particularly at immature stages intercepted in fruit consignments originating from South Africa, is essential but remains an ongoing challenge. A rapid and accurate identification assay to differentiate these five species is needed for inspection and pest surveillance. High throughput sequencing data were generated for each of the five fruit fly species, and five sets of species-specific primers were designed for use in a multiplex PCR. Each primer set amplifies an amplicon of a different size for each species allowing for accurate identification. PCR sensitivity tests demonstrate that the limit of detection for this assay is 10 ng and 4 ng of DNA when extracted from larvae and adult specimens, respectively. The assay developed can be applied in fruit inspection and survey activities within the country and at ports of entry.

## Introduction

Tephritidae is an agriculturally important family with many fruit fly species known to cause extensive damage to commercial fruit^1^. Quarantine restrictions are in place to limit any further spread of these fruit fly pests. In South Africa, five economically important fruit flies are present that can potentially affect the production and export of commercial fruit^2–4^. They are *C. capitata* (Wiedemann), Mediterranean fruit fly; *C. cosyra* (Walker), marula fly; *C. rosa* (Karsch), Natal fly*; C. quilicii* De Meyer, Mwatawala & Virgilio, Cape fly; and *B. dorsalis* (Hendel), the Oriental fruit fly. *Ceratitis quilicii* is a recently described species^3^, hence the pest status and host range of this species in commercial fruit production areas in South Africa are still being determined. The *Ceratitis* species are of Afrotropical origin^5^ while *B. dorsalis* is of Asian origin and was introduced in the northeastern parts of South Africa in 2013^4^. The five fruit fly species are polyphagous (attacking fruit from different plant families)^6^, and two of them, *C. capitata* and *B. dorsalis*, have demonstrated a high affinity for invasiveness with significant expansion of their distribution beyond their native ranges^7,8^. This is a major challenge for horticultural and export industries, particularly with the increasing frequency of international trade^9^. These five fruit flies are currently the only major tephritid pests of commercial fresh fruit produced primarily for export from South Africa.

South Africa is a significant producer and exporter of fresh fruit. In the 2019/2020 production season, over 6.5 million metric tons of fruit were produced, and more than half of the total produce was exported (Fruit South Africa, *2020 Key Fruit Statistics*). Fruit fly pests are of phytosanitary significance for fresh fruit exported from South Africa. It is not uncommon for multiple fruit fly species to infest the same commercial fruit, as the host range of these fruit flies often overlap^10^. South African fruit must meet the country-specific phytosanitary requirements of the export markets to prevent the entry of fruit fly pests^11^. The interception of phytosanitary pests on consignments at Ports of Entry (PoE) can result in the destruction of the commodity or return of the commodity to the country of origin^12^. The time required to accurately identify any pests present in consignments delays the shipment of fresh produce. Fresh fruit and vegetables may be detained for days while undergoing inspection, reducing their economic value. The European Union (EU), an important export market for fresh fruit from South Africa, has zero-tolerance enforcement for non-EU Tephritidae, including all fruit fly pests in South Africa, except *C. capitata*, which is an established pest in the EU^13^. There is a need to be able to distinguish between *C. capitata* and the other four fruit fly pests during inspection before and after export for markets such as the EU. Therefore, a rapid method to accurately identify the five fruit fly pests infesting fresh fruit in South Africa is essential.

Inspection and survey of fruit fly species are often largely reliant on morphological identification of specimens by expert taxonomists and published keys^1,6,14^. The morphological identification of fruit flies to species level can be more reliably made at the adult stage, either emerged adults from infested fruit or adults collected from traps, using these keys. The difficulty arises in differentiating between cryptic species or damaged adult specimens where few distinguishing morphological differences exist, and female specimens appear near identical. However, when immature stages are intercepted, either eggs or larvae in fruit or pupae in soil, and development to adulthood is not practical due to time sensitivity, identification to species level using molecular methods would be more appropriate^15^. There are keys to differentiate between species at the larval stages. Still, these can only be used at the third instar stage and remain problematic if either the specimen is not in good condition, earlier life stages are intercepted, or pupae are found. Larval identification using these keys does not enable the identification of closely related species or species complexes^14^. *Ceratitis rosa* and *C. quilicii* are examples of cryptic species that were previously thought to be the same species until their recent separation in 2016 based on morphological and genetic differences^3,16^. Research has demonstrated that the taxonomic classification of many of these cryptic fruit fly species cannot reliably be resolved through morphological characterization alone, where population-level variation can be easily confused with species-level variation^17^. A molecular-based identification assay may alleviate some of the difficulties experienced in the morphological identification of such closely related species.

Molecular identification tools can offer the advantage of a faster turnaround time as the life stage of the specimen is not a limiting factor. Broad detection assays for tephritid fruit flies have been developed, although they do not reliably allow for identification to species level^18,19^. Microsatellite markers have been considered for identification of closely related fruit fly species, however, this can be expensive and time consuming where six to 16 markers have to be used and unambiguous species identification is not possible without prior morphological identification^20–22^. Molecular identification techniques for fruit flies have been primarily centered around DNA barcoding using cytochrome c oxidase subunit I (COI). Although this method can differentiate between many fruit fly species, it cannot accurately differentiate between species complexes such as FARQ (*C. fasciventris* (Bezzi), *C. anonae* Graham, *C. rosa,* and *C. quilicii)*^3,23,24^ and the *B. dorsalis* complex^25^. Literature suggests that the failure of COI to identify closely related species may be due to incomplete lineage sorting within these species complexes^25^. Misidentifications can be reduced by introducing a distance threshold, where a query sequence is considered unidentifiable if the closest DNA barcode match exceeds the value of the distance threshold set. However, if the distance threshold is too restrictive, it is at the sacrifice of reduced barcoding accuracy with a higher proportion of discarded queries^26^. DNA barcoding relies on time-consuming DNA sequencing, an additional expenditure not applicable for routine analysis^27,28^. COI has also been used for qPCR and real-time PCR identification assays^18,29^. Expansions into other mitochondrial genes for PCR–RFLP analysis and Tephritidae identification have also been explored^27,30^. However, most molecular identification assays based on mitochondrial genes had limitations in identifying closely related species and species complexes.

An investigation into the use of genomic regions as opposed to mitochondrial research for tephritid fruit fly identification was undertaken in this study. The ability to identify multiple species simultaneously and rapidly without the need for costly downstream analysis and sequencing was deemed a priority. Multiplex PCR offers the ability to amplify different DNA targets and different amplicon sizes in a single run. Although the use of multiplex PCR for fruit fly identification has not been well explored, it has shown promising results in differentiating a species of interest, *Rhagoletis cerasi* Loew, from other tephritid flies in North America as well as fruit fly parasitoid identification^31,32^. While the five fruit flies under study can be identified through a variety of existing molecular assays, to date, no assay can identify all five flies simultaneously. Therefore, this study utilizes a multiplex PCR approach to provide a fast and accurate identification assay for differentiation of five tephritid fruit flies of economic importance to South Africa without life-stage restrictions. Many of these tephritid fruit fly species also occur in other parts of Africa^3,5,21,33^. As such the development of a rapid and accurate identification technique in this study will be applicable for fruit fly identification in other parts of Africa where these species occur and are of economic importance.

## Results

### Species identification and DNA extraction

All adult specimens used in this study underwent morphological identification and DNA barcoding targeting the COI gene with the universal primer pair CI-J2183 and TL2-N3014^34^. All adult specimens were identified to species level through morphological identification using published keys^6^. When DNA barcoding was carried out on these specimens, the COI region could only identify *C. capitata*, *C. cosyra*, and *B. dorsalis* to species level. Sequence similarity between *C. rosa* and *C. quilicii* prevented differentiation based upon this gene region. DNA was successfully extracted from each specimen. DNA concentrations ranged from 25.4 to 320.0 ng/µl as determined by a Qubit dsDNA BR assay kit (Invitrogen). The DNA quality determined at the A260/A280 absorbance ratio on a NanoDrop 2000 spectrophotometer ranged between 1.9 and 2.12.

### Gene selection, primer design, specificity, and sensitivity

**Figure 1:**
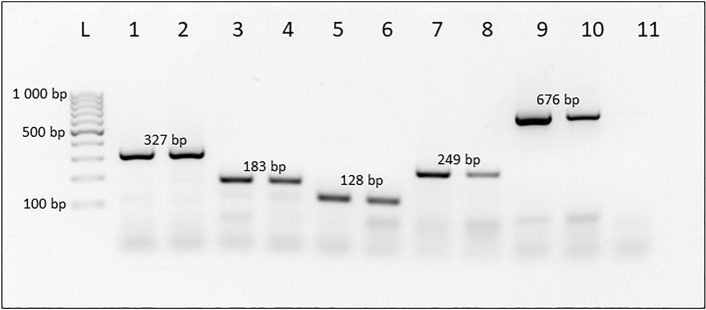
2% agarose-TAE gel displaying the specificity of the multiplex PCR assay on freshly extracted DNA from colony-reared insects with species-specific amplicon size indicated. Lanes 1 & 2: *C. capitata*, Lanes 3 & 4: *C. cosyra*, Lanes 5 & 6: *C. quilicii*, Lanes 7 & 8: *C. rosa*, Lanes 9 & 10: *B. dorsalis*, Lane 11: No template control, Lane L: GeneRuler 100 bp DNA ladder (Thermo Scientific). The original gel is presented in Supplementary Fig. S1a.

**Figure 2:**
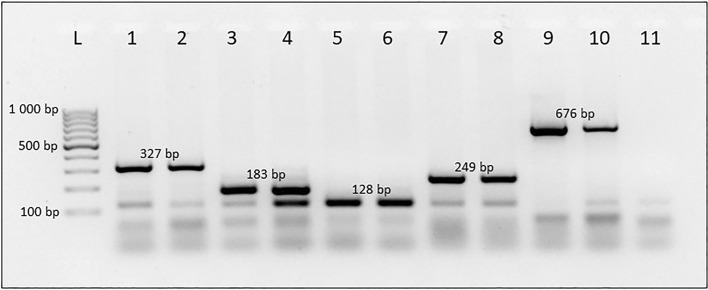
2% Agarose-TAE gel displaying the specificity of multiplex primers in the case of duplex formation in freshly extracted colony-reared larval DNA with species-specific amplicon size indicated. Lane 4 demonstrates the expected *C. cosyra* amplicon at 183 bp with non-specific amplification at 128 bp leading to the formation of a duplex; the larger 183 bp amplicon should be used for identification. Lane 5 is a single 128 bp amplicon indicative of *C. quilicii*. Lanes 1 & 2: *C. capitata*, Lanes 3 & 4: *C. cosyra*, Lanes 5 & 6: *C. quilicii*, Lanes 7 & 8: *C. rosa*, Lanes 9 & 10: *B. dorsalis*, Lane 11: No template control, Lane L: GeneRuler 100 bp DNA ladder (Thermo Scientific). The original gel is presented in Supplementary Fig. S2a.

De novo assembled contigs with high similarity to GenBank accessions XM_004526176.3 and XM_011215866.3 were targeted for species differentiation and primer design. One primer set was designed for each species with differing amplicon lengths for use in a multiplex PCR. Specificity tests performed on freshly extracted DNA from colony-reared insects showed the presence of a single amplicon of the expected size for each species (Fig. 1). The results were consistent when tested on colony larvae, as shown in Fig. 2. A 2% agarose-TAE gel allowed for adequate separation of amplicons that were close in size for accurate species identification. Overall, the assay’s detection limit was 10 ng and 4 ng when tested on colony larvae and colony adult DNA, respectively.

### Assay validation on wild insects

**Figure 3:**
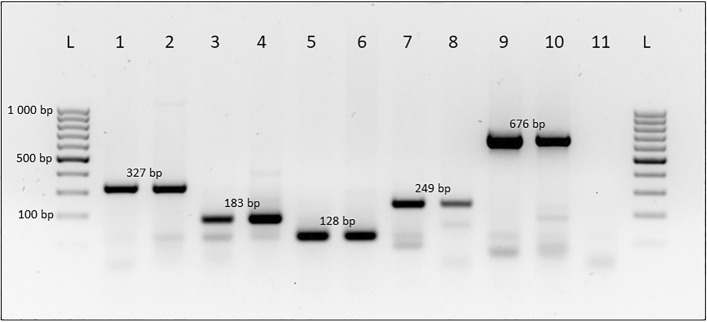
2% agarose-TAE gel displaying the efficacy of the Multiplex PCR assay to identify wild, trap-collected specimens with examples of non-specific amplification. Species-specific amplicon sizes are indicated. Lanes 1 & 2: *C. capitata*, Lanes 3 & 4: *C. cosyra*, Lanes 5 & 6: *C. quilicii*, Lanes 7 & 8: *C. rosa*, Lanes 9 & 10: *B. dorsalis*, Lane 11: No template control, Lane L: GeneRuler 100 bp DNA ladder (Thermo Scientific). The original gel is presented in Supplementary Fig. S3.

The multiplex PCR assay was validated using freshly extracted DNA from wild, trap-collected specimens morphologically identified using available taxonomic keys^6^. It was noted that certain trap-collected specimens produced non-specific amplification of various sizes in addition to the expected identity amplicon. However, none of the non-specific amplicons interfered with the reliability or accuracy of the assay. An example of the efficacy of the multiplex PCR assay on trap-collected fruit flies can be found in Fig. 3.

## Discussion

Five pairs of species-specific primers were designed, and a multiplex PCR was developed to identify fruit flies of economic importance in South Africa to species level. This assay generates a single amplicon of varying sizes for the different fruit fly species, *C. capitata* (327 bp), *C. cosyra* (183 bp), *C. quilicii* (128 bp), *C. rosa* (249 bp), and *B. dorsalis* (676 bp). These amplicons can be separated on a 2% agarose gel allowing for accurate differentiation without downstream analysis and sequencing. DNA concentrations of wild, trap-collected query specimens were not normalized during assay validation to demonstrate the efficacy of this assay for routine identification where concentration normalization is not a priority, saving time when large numbers of specimens are being processed simultaneously.

In this study, all morphologically identified query specimens were correctly identified to species level using the multiplex PCR assay. This assay was developed for use as a differentiation tool to identify fruit fly pests of fresh fruit in South Africa that could potentially be present in export consignments and only validated to identify the five fruit fly pest species currently present in the country. The false-positive rate incurred when other fruit fly species are queried using this assay is unknown. Since other tephritid flies present in South Africa are not pests of commercial fruit primarily exported from South Africa, it is expected that only the five fly species investigated are likely to be intercepted on commercial fruit produced for export.

It was noted that in some specimens, a non-specific amplicon was present at the same size as the expected *C. quilicii* amplicon. The presence of this duplex is not to be confused with the *C. quilicii* amplicon, which will always yield a single 128 bp amplicon with no non-specific amplification. In cases where the duplex is present, the larger amplicon is to be used for species identification (Fig. 2). The species-specific primer pair designed for *C. quilicii* is located just outside of the opsin Rh4 coding domain in an intergenic region which has the potential to cross amplify in closely related species. Although the location of this primer set can lead to false-positive amplification in closely related species when used in conjunction with the other four species-specific primer sets designed in this study in a multiplex, accurate species differentiation of the five flies investigated can be achieved. The universal primer set CI-J2183 and TL2-N3014^34^ can be used to amplify the COI gene, which can be sequenced and queried against the NCBI database for identity confirmation where applicable. It should be noted that CI-J2183 and TL2-N3014 can accurately identify *C. capitata*, *C. cosyra,* and *B. dorsalis* to species level. *Ceratitis capitata* and *Ceratitis caetrata* Munro (Diptera: Tephritidae) have previously been shown to share a high sequence similarity within the COI gene region, which may result in erroneous identification^35^. However*,* the distribution of *C. caetrata* is limited to Kenya, and the fly has never been reported in South Africa^36^. In closely related species and species complexes such as *C. rosa* and *C. quilicii,* this gene region does not allow accurate differentiation^3,23,29^.

While the multiplex PCR assay designed in this study reliably performs its role, the downside of a multiplex is related to the use of PCR itself where the ability of the assay to reliably detect species present relies on the primer binding region to be conserved enough within the species so that any intraspecific variation present in the target region does not hinder amplification leading to false-negative results. This assay was validated with 15 wild insects per species collected from various sites across South Africa (Table S2) and no false-negative results were obtained. False-positive results can also occur where closely related species are highly similar to the target species leading to amplification. Fortunately, false-positive results are avoided in the multiplex assay described as species-specific amplicons are also size specific, so in cases of cross-amplification observed (Fig. 2) where a duplex is formed the larger amplicon is followed for accurate species identification. There is a high potential for false-positive results when other tephritid flies are queried against this assay, however, this assay is intended for use as a differentiation tool for identifying only the five fruit flies investigated.

The multiplex PCR detection assay developed in this study has application in identifying and monitoring agricultural pests of phytosanitary significance, both for pest management and surveillance practices. This relatively low cost and easy to perform assay uses only essential molecular laboratory equipment. It can be used in a standalone format or in conjunction with existing morphological identification techniques for improved accuracy in species identification. A significant advantage of this proposed method is that it allows for identification to species level without the need for downstream analysis. Reliable species identification can be achieved in under two and a half hours post DNA extraction, which significantly reduces the time required for existing molecular identification by DNA barcoding^37^. The increased turnaround time is a considerable advantage for inspection purposes in the implementation of a systems approach reducing the risk of fruit flies before fruit export, for inspections of fruit consignments at ports of entry as well as for early detection of invasive fruit flies such as *B. dorsalis* which is currently absent in several areas in South Africa^38^.

This assay was designed for fruit fly identification in the South African context to facilitate the identification of fruit flies of economic importance. However, given that many of these species also occur in other parts of Africa and are of economic importance in these regions, the assay may be of practical use in these regions as well. Further research will be required to determine the suitability of this assay for fruit fly identification in other African countries where other economically important tephritid flies occur, more specifically other members of the *Ceratitis* FARQ complex (*Ceratitis fasciventris* and *Ceratitis anonae*). Presently, the multiplex PCR assay developed in this study will provide a useful aid in decision-making regarding international trade and for monitoring and detection purposes.

## Methods and materials

### Sample collection, identification, and DNA extraction

Specimens used in this study were stored in 100% ethanol and kept at 4 °C until used. Colony insects and larvae came from established colonies held at Citrus Research International (CRI) in Mbombela, Mpumalanga, South Africa. Detailed information regarding the origin of the colonies is listed in Supplementary Table S1. The identities of the fruit fly species in the colonies (adult specimens from colonies refreshed in the period 2020–2021) were confirmed by Marc De Meyer, Royal Museum for Central Africa, on 21 February 2022. DNA was extracted from single insects following an adapted “salting out” protocol by Sunnucks and Hales^39^, with TNES buffer (50 mM Tris, pH 7.5, 400 mM NaCl, 20 mM EDTA, 0.5% SDS) substituted for 180 µl ATL buffer (Qiagen) and incubation taking place overnight at 56 °C. Following the NaCl precipitation, 2 µl RNAse A was added to the supernatant and the second precipitation took place overnight at − 20 °C with isopropanol. DNA concentration and quality were quantified using a NanoDrop 2000 spectrophotometer and a Qubit dsDNA BR assay kit (Invitrogen).

Wild insects used for validation of the assay were collected from traps. Flies of the genus *Ceratitis* were trapped with McPhail type bucket traps baited with enriched ginger root oil (EGO lure) (Insect Science, Tzaneen, South Africa), and *B. dorsalis* flies were trapped with Chempac bucket traps baited with methyl eugenol (ME) (Invader lure, RiverBioscience, Gqeberha, South Africa). Total DNA was extracted from the whole body of the fruit fly following the destructive protocol of the DNeasy Blood and Tissue Kit (Qiagen).

The species of each adult colony specimen in this study was confirmed before the assay design using universal primer set CI-J2183 and TL2-N3014^34^ for amplification and Sanger sequencing of the COI gene. The PCR was performed in a total volume of 25 µl containing 1 × Kapa Taq buffer A (KAPA Biosystems), 0.2 mM dNTP mix (Thermo Scientific), 0.4 µM of each primer (CI-J2183 and TL2-N3014), and 0.05 U KAPA Taq DNA Polymerase (KAPA Biosystems). The cycling conditions included an initial denaturation step at 94 °C for 5 min, followed by 35 cycles of 94 °C for 30 s, annealing at 50 °C for 30 s and elongation at 72 °C for 45 s. The final extension took place at 72 °C for 7 min.

### High Throughput Sequencing and De novo assembly

DNA from two adult male specimens from the colony of each species were sent for high throughput sequencing at Macrogen (South Korea). Macrogen conducted library construction and high throughput sequencing of the colony insects on the Illumina NovaSeq 6000 platform (2 × 150 bp paired-end reads). Library preparation was performed using the TruSeq DNA PCR-Free Kit for the samples *C. rosa* 1, *C. quilicii* 1 & 2 and *C. cosyra* 2; and the TruSeq Nano DNA Kit for samples *C. capitata* 1 & 2, *C. rosa* 1, *C. cosyra* 1, and *B. dorsalis* 1 & 2, with input ranging from 0.565 to 2.998 µg of genomic DNA. De novo assembly was performed using CLC genomics workbench version 11.0.1 (Qiagen) and SPAdes^40^ using default parameters as well as Velvet^41^ with a hash length of 55.

### Gene selection

Gene regions frequently used for differentiation of insect species were selected from literature and underwent preliminary bioinformatic analyses. A detailed list of these genes is available in Supplementary Table S3. The de novo assembled contigs were queried using BLAST + standalone (BLASTn algorithm) against a local copy of the NCBI GenBank nucleotide database. The gene regions of interest were then identified, and multiple sequence alignments were constructed to compare the genes between species using CLC genomics workbench version 11.0.1 (Qiagen). De novo assembled contigs with high similarity to GenBank accessions XM_004526176.3 and XM_011215866.3 (annotated as Opsin Rh3/Rh4) were targeted for species differentiation and primer design. This gene region showed the greatest potential for species identification due to the number of single nucleotide polymorphisms observed between species in the multiple sequence alignment. Literature suggests that the function of opsins within the order Diptera extends beyond visual processes influencing adaptation to new ecological niches and playing additional roles in host fruit detection, gustatory reception, and taste^42–46^.

### Primer design and multiplex PCR

A multiple sequence alignment of two reference sequences available on GenBank belonging to *C. capitata* and *B. dorsalis*, accessions XM_004526176.3 and XM_011215866.3 respectively, as well as de novo-assembled contigs high in similarity to these reference sequences for each species (GenBank accessions: ON505377–ON505386), was constructed. Five primer sets (IDT) were designed for differentiation of each species by amplicon size using Oligo Explorer 1.1.2 (Gene Link) (Table 1).

**Table 1:** List of primers designed for accurate species identification in the multiplex PCR assay.

Primer pair	Sequence (5’–3’)	Amplicon size (bp)
Opsin4_capitata_F	GCTAAAGCCATAACAATTCAG	327
Opsin4_capitata_R	CAGACTGTTCTTTTGGGC	
Opsin4_cosyra_F	GCTGTGACTTTGTTACAG	183
Opsin4_cosyra_R	GCATACTTGAATCTCAATCGAA	
Opsin4_quilicii_F	GCGTTCTGTTTTTAATCACTCA	128
Opsin4_quilicii_R	CATTTAATGTTTCAGAAGTGCT	
Opsin4_rosa_F	ATTGCTACAACTTTGTCGC	249
Opsin4_rosa_R	GCAGTAATACTGCGAATCATC	
Opsin4_dorsalis_F	TAGCACAATTATTTAGCGGG	676
Opsin4_dorsalis_R	ATTACCGTCAGCGATCAG	

The PCR was performed in a total volume of 25 µl containing 1 × KAPA Taq buffer A (KAPA Biosystems), 0.2 mM dNTP mix (Thermo Scientific), 0.24 µM Opsin4_capitata_F & R, 0.32 µM Opsin4_cosyra_F & R, 0.32 µM Opsin4_quilicii_F & R, 0.64 µM Opsin4_rosa_F & R, 0.64 µM Opsin4_dorsalis_F & R and 0.05 U KAPA Taq DNA Polymerase (KAPA Biosystems). The cycling conditions included an initial denaturation step at 94 °C for 5 min, followed by 35 cycles of 94 °C for 30 s, annealing at 55 °C for 30 s and elongation at 72 °C for 35 s. The final extension took place at 72 °C for 7 min.

All visualizations of multiplex PCR amplicons in this study were separated on a 2% agarose TAE (2 M Tris, 1 M glacial acetic acid, 0.05 M Na_2_EDTA, pH 8) gel stained with ethidium bromide.

To confirm that each specific primer pair generated the expected amplicons, each amplicon was bi-directionally Sanger sequenced with the relevant species-specific primer pair at the Central Analytical Facility of Stellenbosch University. A dilution series with a dilution factor of 5 was made with DNA extracted from both adult and larval colony specimens to determine the assay’s sensitivity. The dilution series was quantified using the Qubit dsDNA BR assay kit (Invitrogen). The multiplex PCR assay was thereafter performed with the dilution series for adult insect DNA (20–0.0064 ng) and for larval DNA (50–0.0000256 ng) to determine the limit of detection.

### Assay validation

The assay was validated using freshly extracted DNA from wild, trap-collected specimens of all five fruit flies, morphologically identified to species level using taxonomic keys. In total the assay was validated on 15 wild fruit flies of each species. 1 µl DNA was taken directly from the extract and used in the multiplex PCR without normalization for DNA concentration.

## Supplementary Information

Supplementary Information.

## Data Availability

The datasets generated and analyzed during this study are available in the NCBI GenBank repository, accession number: ON505377–ON505386.
