# Supplementary material for: A multiplex PCR assay for the identification of fruit flies (Diptera: Tephritidae) of economic importance in South Africa
Source: Sci Rep. 2022 Jul 29;12:13089. doi: 10.1038/s41598-022-17382-x (PMC9338231; doi:10.1038/s41598-022-17382-x)
Supplement: Supplementary file 1 — Supplementary Information. [file 41598_2022_17382_MOESM1_ESM.pdf]

A Multiplex PCR assay for the identification of fruit flies (Diptera: Tephritidae) of economic importance in South Africa

Kelsey J Andrews[1], Rachelle Bester[1,2], Aruna Manrakhan[3,4], and Hans J Maree[1,2,*]

Supplementary material:

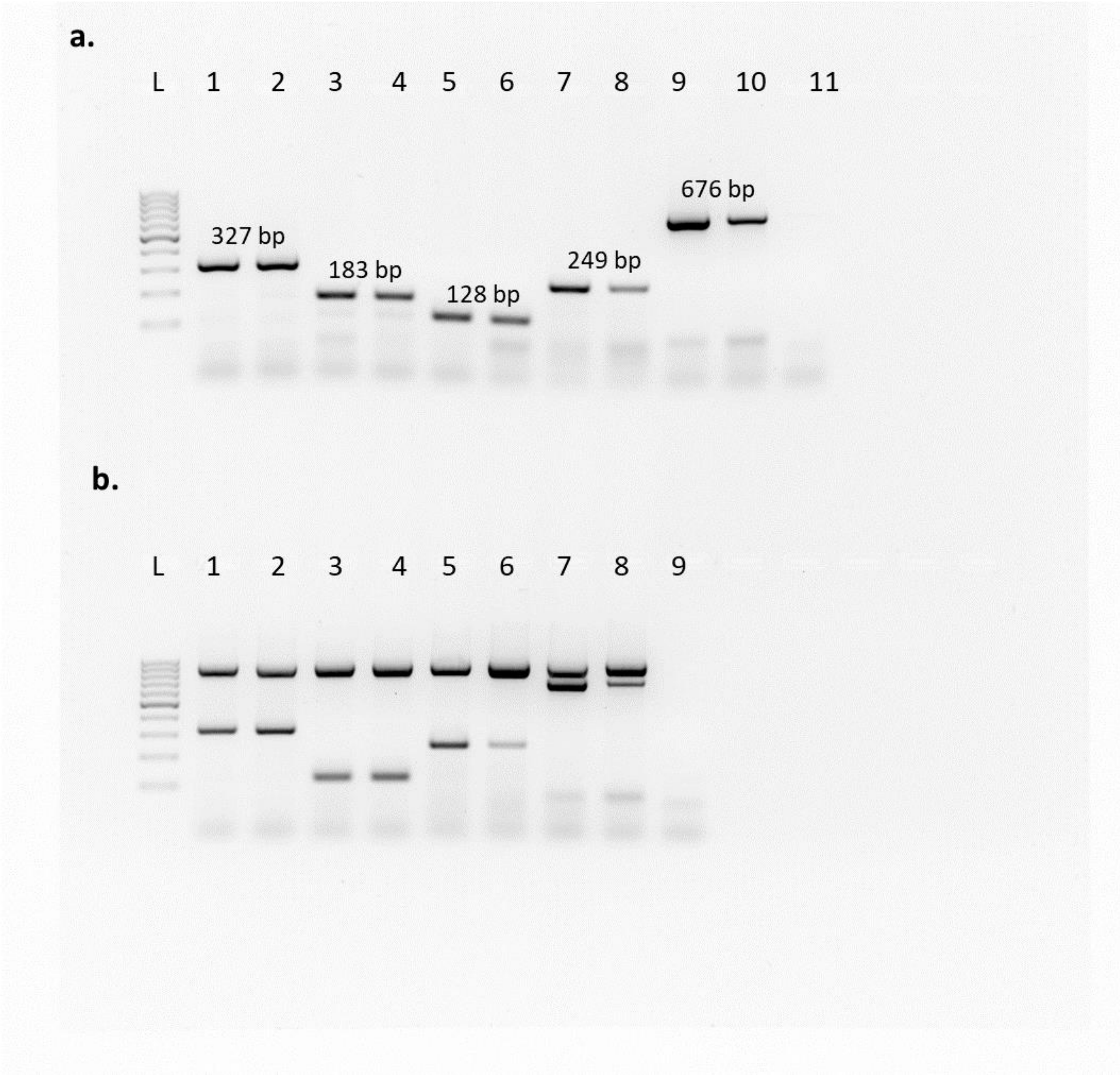

Figure S1. (a.) 2% agarose-TAE gel displaying the specificity of the multiplex PCR assay on freshly extracted DNA from colony-reared insects with species-specific amplicon size indicated. Lanes 1 & 2: *C. capitata*, Lanes 3 & 4: *C. cosyra*, Lanes 5 & 6: *C. quilicii*, Lanes 7 & 8: *C. rosa*, Lanes 9 & 10: *B. dorsalis*, Lane 11: No template control, Lane L: GeneRuler 100 bp DNA ladder (Thermo Scientific). **(b.)** 2% agarose-TAE gel displaying the multiplex PCR (excluding *C. cosyra* primers) in conjunction with the universal primer set CI-J2183 and TL2-N3014 amplifying the COI gene in colony reared insects. Lanes 1 & 2: *C. capitata*, Lanes 3 & 4: *C. quilicii*, Lanes 5 & 6: *C. rosa*, Lanes 7 & 8: *B. dorsalis*, Lane 9: No template control, Lane L: GeneRuler 100 bp DNA ladder (Thermo Scientific).

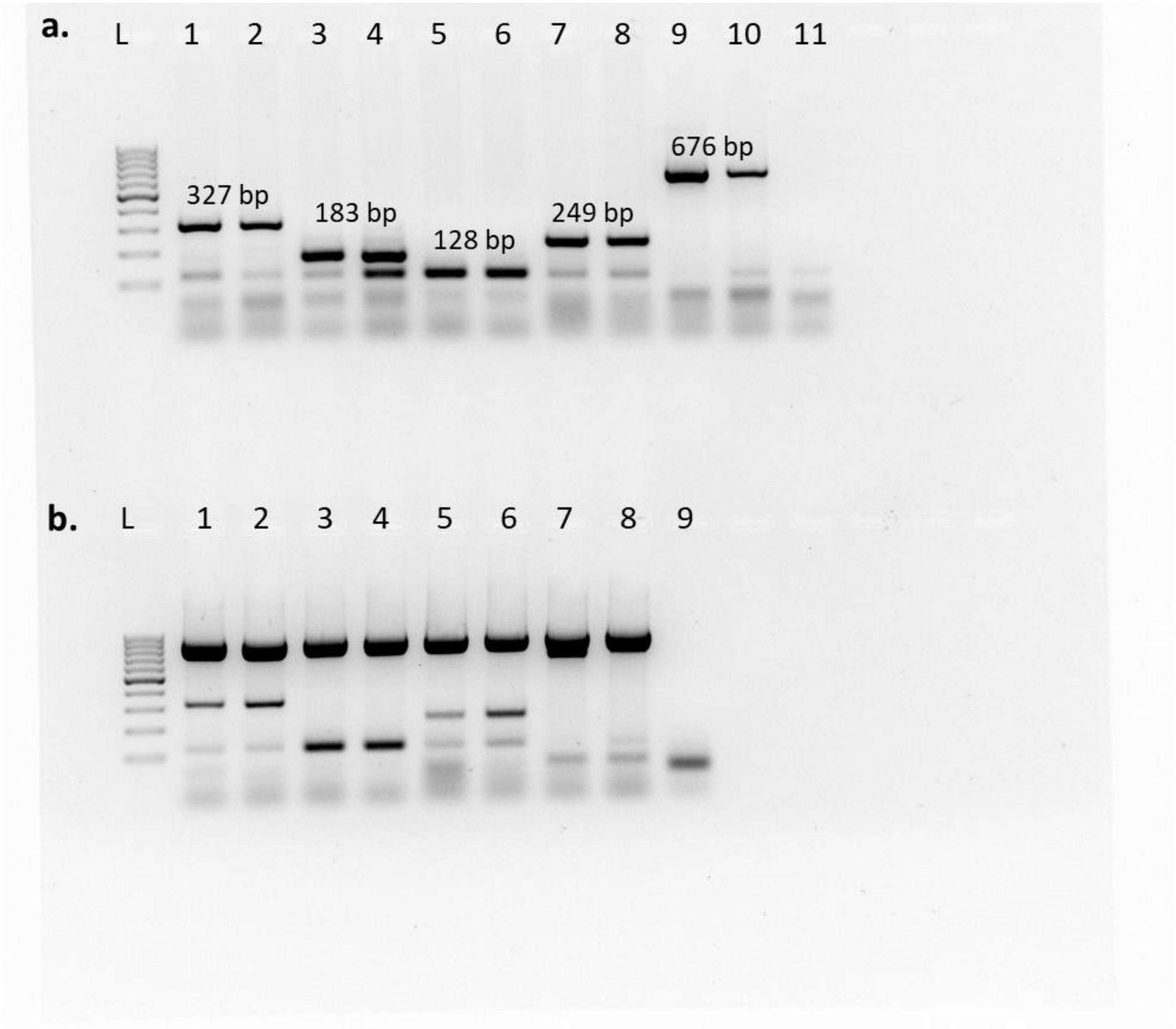

Figure S2 (a.). 2% Agarose-TAE gel displaying the specificity of multiplex primers in the case of duplex formation in freshly extracted colony-reared larval DNA with species-specific amplicon size indicated. Lane 4 demonstrates the expected *C. cosyra* amplicon at 183 bp with non-specific amplification at 128 bp leading to the formation of a duplex; the larger 183 bp amplicon should be used for identification. Lane 5 is a single 128 bp amplicon indicative of *C. quilicii*. Lanes 1 & 2: *C. capitata*, Lanes 3 & 4: *C. cosyra*, Lanes 5 & 6: *C. quilicii*, Lanes 7 & 8: *C. rosa*, Lanes 9 & 10: *B. dorsalis*, Lane 11: No template control, Lane L: GeneRuler 100 bp DNA ladder (Thermo Scientific). **(b.)** 2% Agarose-TAE gel displaying the multiplex PCR (excluding *C. cosyra* primers) in conjunction with the universal primer set CI-J2183 and TL2-N3014 amplifying the COI gene in colony reared larvae. Lanes 1 & 2: *C. capitata*, Lanes 3 & 4: *C. quilicii*, Lanes 5 & 6: *C. rosa*, Lanes 7 & 8: *B. dorsalis*, Lane 9: No template control, Lane L: GeneRuler 100 bp DNA ladder (Thermo Scientific).

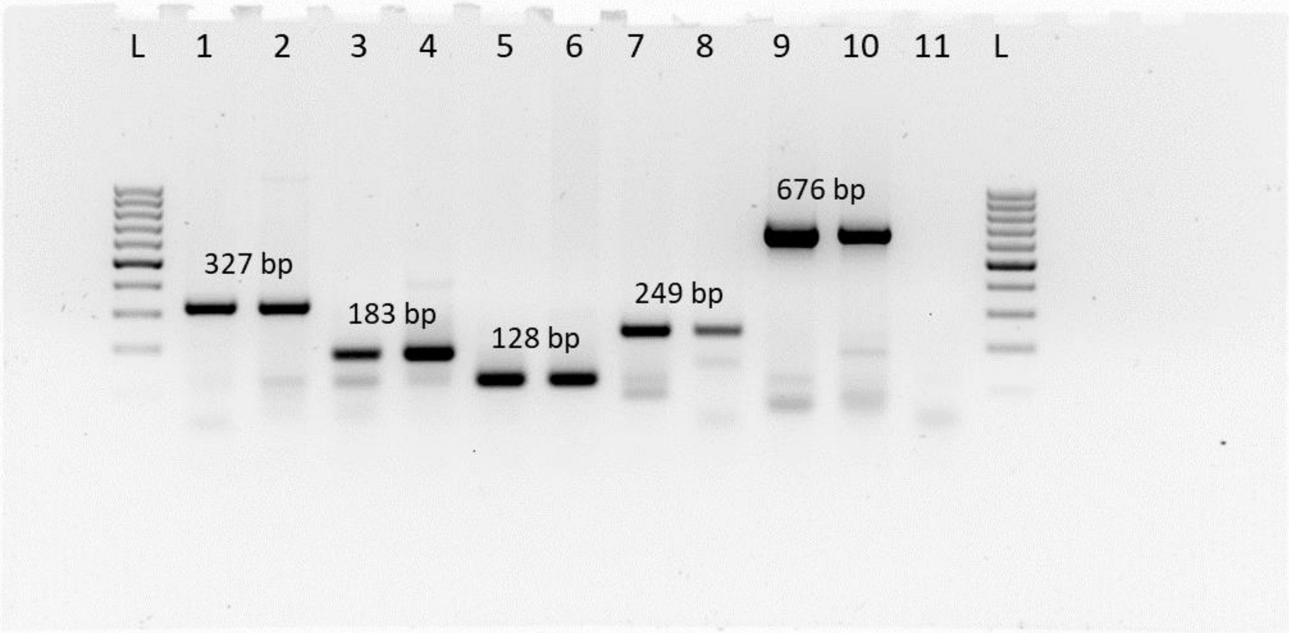

Figure S3. 2% agarose-TAE gel displaying the efficacy of the Multiplex PCR assay to identify wild, trap-collected specimens with examples of non-specific amplification. Species-specific amplicon sizes are indicated. Lanes 1 & 2: *C. capitata*, Lanes 3 & 4: *C. cosyra*, Lanes 5 & 6: *C. quilicii*, Lanes 7 & 8: *C. rosa*, Lanes 9 & 10: *B. dorsalis*, Lane 11: No template control, Lane L: GeneRuler 100 bp DNA ladder (Thermo Scientific).

Life stage	Species	Rearing fruit		Collection Date	Latitude	Longitude
Adult male	*C. capitata*	Coffee	(*Coffea canephora Pierre ex. Froehner)*	10/03/2019	31°5’15.63” E	25°6’43.50” S
	C. cosyra	Marula	(*Sclerocarya birrea* (A. Rich.) Hochst.)	1/29/2019	31°2’35.70” E	25°28’4.03” S
	C. quilicii	Peach	(*Prunus persica* L. Batsch)	1/21/2020	30°23’34.73” E	24°59’47.17” S
	C. rosa	Strawberry Guava	(*Psidium cattleyanum* Sabine)	11/13/2019	30°58’10.99’’ E	25°27’08.54’’ S
	B. dorsalis	Mango	(*Mangifera indica* L.)	1/29/2019	30°58’10.99’’ E	25°27’08.54’’ S
Larvae	*C. capitata*	Coffee	(*Coffea canephora Pierre ex.* Froehner)	3/10/2021	31°5’15.63” E	25°6’43.50” S
	C. cosyra	Pepper-bark tree	(*Warburgia salutaris* (Bertol.f.) Chiov.)	11/30/2020	30°58’6.10” E	25°26’37.92” S
	C. quilicii	Pineapple guava	(*Feijoa sellowiana* (O.Berg) O.Berg)	05/2021	29°59’ 11.56” E	26°30’51.31” S
	C. rosa	Jambos	(*Syzygium jambos* L. Alston)	11/27/2020	30°58’10.99’’ E	25°27’08.54’’ S
	B. dorsalis	Mango	(*Mangifera indica* L.)	2/12/2021	30°57’ 15.84” E	25°32’58.06” S

Table S1. Origin of colony flies reared at the CRI (Mbombela, Mpumalanga, South Africa). Adult colony insects collected in February 2021 were refreshed between January 2019 and January 2020. Larval specimens collected in August 2021 were refreshed between November 2020 and May 2021. Collection sites are provided as coordinates.

Species	Collection date	Province	Coordinates
B. dorsalis	08/2021	Mpumalanga	31°04’17.41”E<br>25°26’38.27”S
	07/2021	Mpumalanga	30°34’31.48”E<br>25°23’52.07”S
	06/2021	Limpopo	30°50’51.95”E<br>24°24’26.61”S
	09/2021	Limpopo	30°32’36.02”E<br>23°45’18.95”S
	06/2021	Limpopo	30°22’54.60”E<br>23°52’21.32”S
C. quilicii	06/2021	Northwest	25°46’12.9”S<br>27°36’51.7”E
	06/2021	Mpumalanga	25°22’50.7”S<br>30°32’02.2”E
	06/2021	KwaZulu Natal	27°21’44.7”S<br>31°47’ 14.4”E
	03/2021	Free State	28°15’09.1”S<br>28°19’02.E
	06/2021	Eastern Cape	33°36’43.4”S<br>25°39’39.1”E
C. capitata	06/2021	Northwest	25°46’12.9”S<br>27°36’51.7”E
	06/2021	Limpopo	23°51’47.7”S<br>30°23’08.4”E
	06/2021	Mpumalanga	25°26’39.3”S<br>31°33’15.4”E
	06/2021	KwaZulu Natal	27°21’44.7”S<br>31°47’ 14.4”E
	05/2021	Northern Cape	28°48’8.83”S<br>20°39’56.2”E
C. cosyra	06/2021	Northwest	25°46’12.9”S<br>27°36’51.7”E
	06/2021	Limpopo	23°51’47.7”S<br>30°23’08.4”E
	06/2021	Mpumalanga	25°24’34.3”S<br>30°55’46.6”E
	08/2021	KwaZulu Natal	27°21’44.7”S<br>31°47’14.4”E
C. rosa	06/2021	Mpumalanga	25°26’44.9”S<br>30°58’05.1”E

Table S2. Sample collection data for the wild, trap-collected specimens used for assay validation in this study. The collection site is provided as the province and coordinates

Gene region investigated	Reference sequence *C. capitata*	Reference sequence *B. dorsalis*	Literature cited
Cytochrome c oxidase subunit I	MW410928.1	MZ621836.1	[1,2]
Cytochrome c oxidase subunit II	NC_000857.1	KT343905.1	[1,2]
Protein MTO1 homolog	XM_004517805.4	XM_011208269.2	
Glutamine synthetase 1, mitochondrial	XM_004530302.3	XM_011209495.2	[3]
Presequence protease, mitochondrial	XM_004535774.4	XM_019991906.1	[4]
ERF3A	GAMC01016820.1	GAKP01021067.1	[5]
Dynamin	GAMC01011620.1	XM_011213658.3	[6]
TipE	XM_012302544.2	XM_019992132.1	[7]
Gustatory receptor for bitter taste 22e-like	XM_023303386.1	XM_011212444.1	[8]
Gustatory and pheromone receptor 32a	XM_004526635.3	XM_019991446.1	
CCR4-NOT transcription complex subunit 6-like	XM_012300675.2	XM_019990104.1	
Toll-like receptor Tollo	XM_004522201.2	XM_011209703.2	
Gawky	XM_020860629.1	XM_011203056.2	
Opsin Rh2	XM_004534311.3	XM_011205152.2	
Opsin Blue Sensitive	XM_004525726.3	N/A	
Opsin Rh4	XM_004526176.3	XM_011215866.2	
Opsin Rh1	XM_004535527.3	XM_018947745.1	
Opsin Rh6	XM_004518077.2	XM_011209950.2	

Table S3. List of gene regions utilized in the preliminary bioinformatic analysis, including accession numbers of the reference sequences and the literature from which they were selected.

References:

1. Garzón-Orduña, I. J., Geib, S. M., Ledezma, L., Bremer, F. T. & Barr, N. B. Implementing Low-Cost, High Accuracy DNA Barcoding From Single Molecule Sequencing to Screen Larval Tephritid Fruit Flies Intercepted at Ports of Entry. *Ann. Entomol. Soc. Am.* **113**, 288–297 (2020).

2. Armstrong, K. F. & Ball, S. L. DNA barcodes for biosecurity: invasive species identification. *Philos. Trans. R. Soc. B Biol. Sci.* **360**, 1813–1823 (2005).

3. Killer, J. *et al.* Glutamine synthetase type I (glnAI) represents a rewarding molecular marker in the classification of bifidobacteria and related genera. *Folia Microbiol. (Praha).* **65**, 143–151 (2020).

4. Alikhani, N. *et al.* Targeting Capacity and Conservation of PreP Homologues Localization in Mitochondria of Different Species. *J. Mol. Biol.* **410**, 400–410 (2011).

5. Tarasov, O. V., Zhuravleva, G. A. & Abramson, N. I. Evaluation of the gene encoding translation termination factor eRF3 as a possible phylogenetic marker. *Mol. Biol.* **42**, 834–842 (2008).

6. Hardy, N. B. Phylogenetic utility of dynamin and triose phosphate isomerase. *Syst. Entomol.* **32**, 396–403 (2007).

7. Bourdin, C. M. *et al.* Molecular and functional characterization of a novel sodium channel TipE-like auxiliary subunit from the American cockroach *Periplaneta americana*. *Insect Biochem. Mol. Biol.* **66**, 136–144 (2015).

8. Papanicolaou, A. *et al.* The whole genome sequence of the Mediterranean fruit fly, *Ceratitis capitata* (Wiedemann), reveals insights into the biology and adaptive evolution of a highly invasive pest species.

Genome Biol. **17**, 192 (2016).
